# Hybrid Method of Automated EEG Signals’ Selection Using Reversed Correlation Algorithm for Improved Classification of Emotions †

**DOI:** 10.3390/s20247083

**Published:** 2020-12-10

**Authors:** Agnieszka Wosiak, Aleksandra Dura

**Affiliations:** Institute of Information Technology, Lodz University of Technology, Wólczańska 215, 90-924 Łódź, Poland; aleksandra.dura@dokt.p.lodz.pl

**Keywords:** emotion recognition, EEG electrodes selection, feature selection, the Reversed Correlation Algorithm, data analysis, electromagnetic sensing, machine learning

## Abstract

Based on the growing interest in encephalography to enhance human–computer interaction (HCI) and develop brain–computer interfaces (BCIs) for control and monitoring applications, efficient information retrieval from EEG sensors is of great importance. It is difficult due to noise from the internal and external artifacts and physiological interferences. The enhancement of the EEG-based emotion recognition processes can be achieved by selecting features that should be taken into account in further analysis. Therefore, the automatic feature selection of EEG signals is an important research area. We propose a multistep hybrid approach incorporating the Reversed Correlation Algorithm for automated frequency band—electrode combinations selection. Our method is simple to use and significantly reduces the number of sensors to only three channels. The proposed method has been verified by experiments performed on the DEAP dataset. The obtained effects have been evaluated regarding the accuracy of two emotions—valence and arousal. In comparison to other research studies, our method achieved classification results that were 4.20–8.44% greater. Moreover, it can be perceived as a universal EEG signal classification technique, as it belongs to unsupervised methods.

## 1. Introduction

Bioinformatics techniques for EEG signal analysis help in increasing the diagnostic potential of sleep disorders and detecting diseases of the nervous system. These include epilepsy or depression [1]. Moreover, there has been a growing interest in encephalography to enhance human–computer interaction (HCI) and develop brain–computer interfaces (BCIs) [2]. For BCIs, there are various applications, and their design depends on the intended application. Two main branches of BCI applications can be distinguished—control and monitoring. Control applications are designed to use brain signals to manipulate an external computer, while monitoring applications allow for the user’s mental and emotional state recognition to control the environment in which they are in, or the interface they use [3].

Emotions play an essential role in everyday life and interpersonal communication. Positive emotions enhance health, while negative emotions may contribute to the aggravation of health problems. Research in neurobiology and psychology confirms that emotions constitute a vital factor influencing rational behavior. Furthermore, patients with emotional disorders have difficulty carrying out daily activities [4].

Emotions are identified by verbal (e.g., emotional vocabulary) or non-verbal signals such as gestures, expressions, and intonation [5,6,7]. EEG signals are one of the methods of emotional state identification [8,9]. It is non-invasive and relatively inexpensive in comparison to other signal acquisition methods. However, during semantic EEG signal analysis in terms of mental task classification, the accuracy is still one of the significant problems. Therefore, recognizing emotions is critical for the current research within EEG signal analysis [10,11].

The EEG test records electrical patterns in the brain. During EEG examinations, sensors (electrodes and wires) are placed on the scalp of a patient. The sensors record potentials from the synchronous work of groups of cerebral cortical neurons. Thus, the recordings’ analysis depends on the appropriate placement of the measuring electrodes. Although the measured signal is amplified, retrieving information from the EEG signal is a difficult task. The complexity arises due to both noise from the internal (physiological) and external (hardware related) artifacts, and physiological interferences.

The improvement in signal quality can be achieved by eliminating artifacts, such as [12,13,14,15,16]:

- electrooculography (EOG), which occurs as a result of motion generated by eyes,
- electrocardiogram (ECG), which occurs as a result of heartbeats, and
- electromyogram (EMG), which occurs as a result of muscular motion.

On the other hand, the appropriate selection of features that should be taken into account can enhance the process of EEG signal analysis. Hence, the automatic feature selection of EEG signals is an important research area.

Two approaches can be distinguished within the undertaken research works:

- optimal selection of electrodes [17],
- optimal extraction and selection of features [18].

The optimal selection of measuring electrodes is the first and crucial stage of works related to the automatic selection of EEG signals’ features [19]. However, due to the complexity of the electrode selection and the need to adapt the research to the classification problem, the attempts made so far have not clearly indicated the number and the position of electrodes best suited to the tasks related to emotions’ classification. For example, the study [20] suggests using nine electrodes, while the authors of the research [17] indicate three to eight electrodes depending on the experiment. An additional difficulty is that the smaller sets of electrodes do not constitute subsets of the covering sets of electrodes in the cited works. The selected electrodes proposed in studies [20,21] are located over the frontal and parietal lobes, whereas [17] indicates mainly central and occipital lobes.

The extraction and selection of features also play an essential role in EEG applications. The most commonly used features can be divided into four categories—time domain, parametric model-based, transformed domain, and frequency-based components [19]. Moreover, the features extracted from the EEG may be redundant or contain outliers that degrade the classification performance [22]. To cope with these problems, using feature selection is recommended.

Our proposed research aims to identify a set of key electrodes for the emotional state classification based on the EEG signal as a continuation of research on the optimal selection of electrodes. Most of the earlier strategies focus on correlations between a particular type of emotion and a brain region with a subset of electrodes [10,23]. However, there is still a lack of more flexible solutions independent from emotion type, which perform well for different emotional categories. We propose using a reversed correlation-based algorithm RCA for the automatic selection of electrodes. The RCA was described in [24] and used for medical diagnosis support. Unlike most of the feature selection methods, the RCA technique is an unsupervised learning method. It implies the possibility of selecting a set of channels independent from the classified emotion. Moreover, the RCA significantly reduces the data necessary for further analysis while maintaining high classification accuracy.

The remainder of the paper is organized as follows. In the next section, related works, considering different techniques of emotion recognition, are presented. Then, the emotions’ classification problem in terms of the EEG test is introduced, and the whole methodology is described. Next, the experiments carried out are depicted, and the obtained results are discussed. The final section presents the study’s conclusions and delineates future research.

## 2. Related Work

Emotions play a vital role in human communication. However, most new human–computer interaction (HCI) systems, which are assumed to signify human interaction, suffer from a lack of emotional intelligence, and are deficient in interpreting emotions. Therefore, many types of research are undertaken to fill this gap and to automate emotional responses.

Various scales of emotions’ categorization were proposed [10]:

- the six basic emotions proposed by Ekman and Friesen [25],
- the tree structure of emotions proposed by Parrott [26],
- Plutchik’s emotion wheel [27], and
- the valence–arousal scale by Russell [28].

**Figure 1:**
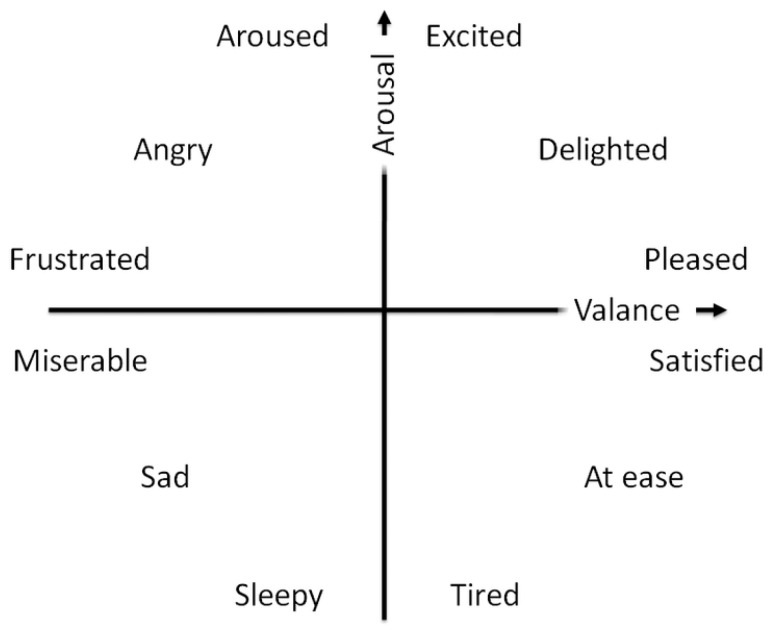
Russell’s circumplex model of affect.

Russell’s valence–arousal scale is one of the most widely used scales [28]. In this scale, each psychological state is placed on a two-dimensional plane with arousal and valence as the vertical and horizontal axes. As it was depicted in Figure 1:

- arousal ranges from inactive (bored and uninterested) to active and excited,
- valence spreads from unpleasant (sad and stressed) to happy and pleasant.

In the past decades, many researchers have investigated subjects’ reactions and potentials of different emotion recognition techniques, including speech, non-verbal audition, facial expression, visual and thermal images, peripheral neural signals, and central neural system signals [29,30,31,32,33,34,35,36,37].

Infrared thermography (IRT) captures the radiation of energy from the use of sensors. Investigations revealed that there is a connection between emotions and blood flow, and such a connection can be recognized using infrared thermography [32,33,34].

The authors of [32] aimed at classifying the main emotions—joy, disgust, anger, fear, and sadness—using facial temperature reactions. Their system estimates changes in temperature from the face’s central area (forehead, cheeks, nose, and maxillary) and assigns the approrpiate emotion. The accuracy of the classification was 89.9%. The first three emotions—joy, disgust, and sadness—showed great accuracy, at 96.96%, 91.73%, and 89.33%, respectively.

Another method using landmark detection in thermal images was proposed in [33]. The main goal of these studies is to receive the best accuracy for emotion recognition by child–robot interaction. This kind of robotic system can be used as a therapy-aid and help children who suffer from autism and have problems with showing emotions. The researchers used various methods for feature extraction and reduction (algorithm Viola-Jones, PCA, LDA). They attained mean accuracies of 85.75% and 81.84% for the main five emotions.

The authors of [34] proposed a new algorithm for emotion recognition by analyzing facial thermal images. The algorithm divides an image into grids and predicts the probabilities of emotions for each part of the grid. Their study indicated that background has an impact on the results, and it is important to eliminate the surrounding, except for the face. Seven emotions—sadness, happiness, surprise, fear, anger, neutral, and disgust—were trained and tested. The average accuracy of all emotions was 65%. However, the accuracy depended on the emotions. The best result of 80.8% was attained for happiness. Emotions such as sadness, surprise, and disgust were above 50% accuracy. However, for anger, fear, and neutral emotions, the accuracies were less than 30%.

Some of the research on thermal images have satisfactory results. However, there is still an insifficient number of exhaustive studies about some of the emotions, which could improve quality. The main problem is the lack of datasets with thermal images, which prevent the development of emotion detection using infrared thermography. The other drawback is the requirement of expensive devices, such as a special infrared camera [32].

Another method for emotion detection is by speech. The authors of [35] created a new database to conduct research on emotion recognition by speech, which consists of basic emotions—neutral, anger, happiness, sadness, disgust, surprise, and fear. The accuracy was enhanced to 78.89% compared to the renowned EMO database −74.72%.

Zhu et al. [36] took into account a combination of the Support Vector Machine (SVM) and a Deep Belief Network (DBN) in emotion detection from Chinese speech. Their method consisted of two steps. First, features were extracted, and the algorithm selected only those with the highest importance for classification. The second step was to find the best way for emotion recognition. The authors introduced a novel approach, which joined two algorithms—DBN and SVM. Their experiments resulted in a great accuracy of 95.6%. However, the accuracies of using SVM and DBN separately were 84.6% and 94.6%, respectively, so the authors’ hybrid method improved on the previous results.

The authors of [37] introduced a Deep Convolutional Neural Network (DCNN) during feature selection. They used several classification methods—support vector machine, random forest, the k-nearest neighbors’ algorithm, and neural network classifiers. The average accuracy for all experiments achieved 85.58%. The authors also reported that DCNN models are efficient for feature extraction during speech and can bring a noticeable improvement in emotion recognition by speech.

According to neurophysiological and clinical research, electroencephalogram (EEG) signals reflect the brain’s electrical activity and the mind’s functional condition. Therefore, numerous supervised and unsupervised approaches based on EEG signals have been proposed in many applications, including motor imagery or epileptic seizure detection. EEG signals also express the human emotional state, and in the last several years, EEG signals have been slowly introduced to emotion recognition due to their strong objectivity, and moderately good classification accuracy [38].

The main goal of many investigations has been to identify brain areas and patterns associated with emotional states. Most of the studies stress high reactivity of negative emotions [39,40] and their relations to right-brain activity [41].

EEG data are accumulated from many locations across the brain and frequently entail data from more than 100 electrodes. That is why efficient channel selection is very significant. The main objective of employing channel selection is to reduce computational complexity while assessing EEG signals without losing classification accuracy. The literature describes that similar or even the same performance could be accomplished by using a more compact group of channels [23,42]. However, researchers still argue about the number and locations of the EEG electrodes [17,20,43]. Therefore, more comprehensive studies on channel selection are of great importance.

Band frequency selection and feature selection are other vast areas of research related to EEG signal analysis. Many works focus on motor imagery (MI) in automated selection techniques, as MI is one of the most common mental tasks used in BCI applications. The subjects are directed to imagine themselves performing a specific motor action, such as moving a foot or hand, without activating any muscles. The authors of [44] introduced a novel system for motor imagery detection. Joining empirical wavelet transform and several algorithms for dimensionality reduction yields a significant increase in performance. The attained accuracy equaled 92.9%. Those studies also undertook and resolved the problem with obtaining good results for small samples.

The BCIs interactions with subjects are one of the major problems described in studies on motor imagery. Electrode selection may be crucial for overcoming interaction problems. In the work [45], the authors explored the reduction of the time-response in motor imagery using four electrodes. The accuracy of 83.8% for detecting hand motion imagery was achieved in a time of 2 s. This research can help a disabled or immobile person communicate with a computer or a wheelchair.

The robustness and computational load are the key challenges in motor imagery based on EEG signals. In [46] Sadiq et al. proposed a multivariate empirical wavelet transform (MEWT)-based method to obtain robustness against noise. The average sensitivity, specificity, and classification accuracy of 98% were achieved by employing multilayer perceptron neural networks, a logistic model tree, and least-squares support vector machine (LS-SVM) classifiers, resulting in an improvement of up to 23.50% in classification accuracy compared with other existing methods [47,48]. The proposed method also shows a great potential in EEG signal processing for emotion recognition.

Electroencephalography is also used for epileptic seizure detection. Epilepsy is one of the most common brain disorders and makes life difficult for many people [49]. The work [50] introduced a novel method for the recording of epileptic signals for EEG. They found that EOG artifacts were not visible during the attack for channels that were located behind the ears. The investigations revealed 38 seizures by the behind-the-ear method among all 47 trials. Detecting epilepsy only thanks to a few channels behind the ears, which are not visible to other people, may render many lives easier.

High accuracy (99.25%) of epileptic seizure detection was achieved by the authors of [51]. They developed a new framework to detect epilepsy rapidly. Wang et al. used various feature extraction, nonlinear analysis of EEG signals, and Principal Component Analysis (PCA).

The research of [52] shows that one of the main points in epileptic seizure detection is finding the most relevant feature. The authors proposed using a graph eigen decomposition (GED)-based approach, which reduces unnecessary attributes. Their method, in conjunction with a feedforward neural network (FfNN), scored 99.55% for accuracy. It is one of the highest results among any other investigations related to epileptic seizure detection by EEG signals.

The authors of the study [53] propose emotion recognition with an improved particle swarm optimization for feature selection. They use an initial set of features from time, frequency, and time-frequency domains and a modified particle swarm optimization (PSO) method with multi-stage linearly-decreasing inertia weight (MLDW) for feature selection. The average accuracy of four-class emotion recognition reached over 76%. Their study also opens other issues that can be considered in the future, such as the optimization of parameter settings.

The research analysis shows that there are many obstacles to overcome in EEG data analysis. In our study, we focus on the EEG electrode selection problem applied to emotion recognition.

## 3. Materials and Methods

### 3.1. The Method Overview

The considered method for indicating the EEG electrodes’ number and locations consists of two main steps—selecting bands and electrodes. They are preceded by one initial step, which aims at adjusting the original dataset to analysis needs, and one final step for evaluation. Therefore, the proposed steps can be presented as follows:

1. EEG data preprocessing, based on the initial signal’s downsampling and applying the bandpass filtering,
2. selecting bands of frequencies based on calculating average frequencies for each second of a trial,
3. selecting electrodes based on a statistical analysis of correlation coefficients,
4. evaluation of emotions’ classification results in terms of valence and arousal.

**Figure 2:**
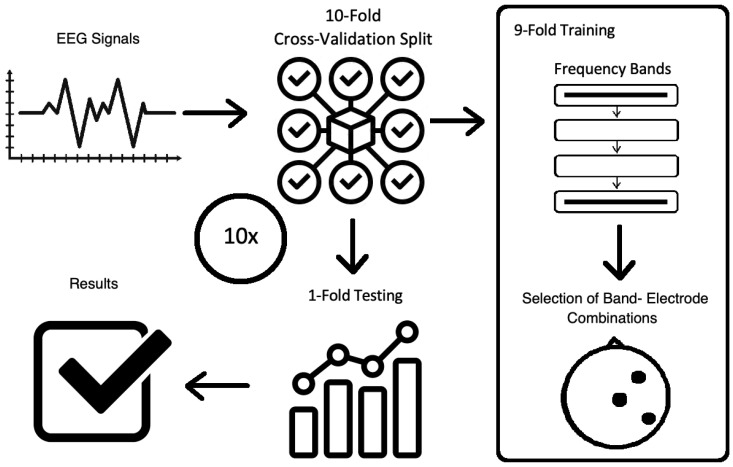
The overview of the methodology.

The general overview of the method is shown in Figure 2. The description of EEG data, as well as the mains steps of the methodology, are presented in Section 3.2, Section 3.3, Section 3.4 and Section 3.5.

### 3.2. EEG Dataset

The experimental study was carried out on the reference DEAP dataset [10], created by researchers at Queen Mary University of London. It is among the most frequently used open-source datasets in research studies. It contains multiple physiological signals with the psychological evaluation. In the experiment, 32 healthy subjects took part, aged between 19 and 37 (50% female, 50% male). Each subject watched 40 one-minute music videos. The process of conducting the experiment for one subject consisted of the following steps:

1. displaying number of the next trial (2 s),
2. baseline recording (3 s),
3. displaying one video (60 s),
4. rating four emotions (valence, arousal, dominance, liking) by each user.

The data comes from 32 electrodes placed on the scalp and 13 physiological electrodes on the examined person’s fingers and face. The electrodes recorded bioelectrical signals while subjects were seeing 40 trials of music movies with different psychological tendencies.

The electrodes pick up values from the four main parts of the brain:

1. the frontal lobe, which is responsible for thinking, memory, evaluation of emotions and situations,
2. the parietal lobe, located just behind the frontal part and responsible for movement, recognition, a sensation of temperature, touch, and pain,
3. the occipital lobe, responsible for seeing and analyzing colors and shapes, and
4. the temporal lobe, located in the lateral parts, and responsible for speech and recognition of objects.

**Figure 3:**
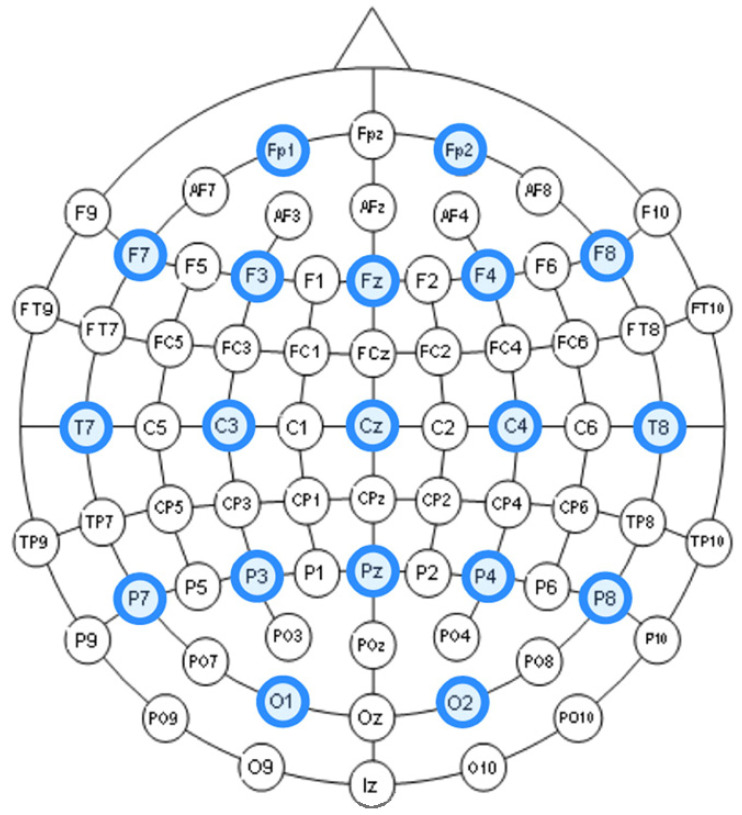
Names of channels.

The entire distribution of channels is shown in Figure 3.

We applied the initial 32-channel EEG signals in the DEAP dataset in our experiments, preprocessed by downsampling to 128 Hz and segmented into a 60 s trial and a 3 s pre-trial baseline. The artifacts of eye movement (EOG) were removed using a blind source separation technique [10].

Four psychophysical states were assessed (valence, arousal, dominance, liking) while viewing 1 min movies in the DEAP dataset. Each video lasted 60 s, and 3 s were reserved for preparing the person for the next trial. Each movie has been ranked on a scale of 1–9. The smallest value indicates negative emotion and higher—strong, positive emotion.

Our research focused on the two most objective feelings—valence and arousal. The valence reflects what the person feels while viewing the film (happy or sad), while arousal demonstrates the degree of the impression the film makes (calm, enthusiasm). The evaluation between 1 and 9 was transformed into a binary scale:

- 0 suggests low valence/arousal, less than 4.5 points, and
- 1 for high valence/arousal, more than 4.5 points.

A score of 4.5 points was designated as a threshold.

### 3.3. Band Selection

There are five widely recognized brain bands—delta (δ), theta (θ), alpha (α), beta (β), and gamma (γ). The main frequencies of human EEG waves, along with their characteristics, are listed in Table 1 [54,55].

For valence and arousal classification, the delta band can be excluded from analysis.

**Figure 4:**
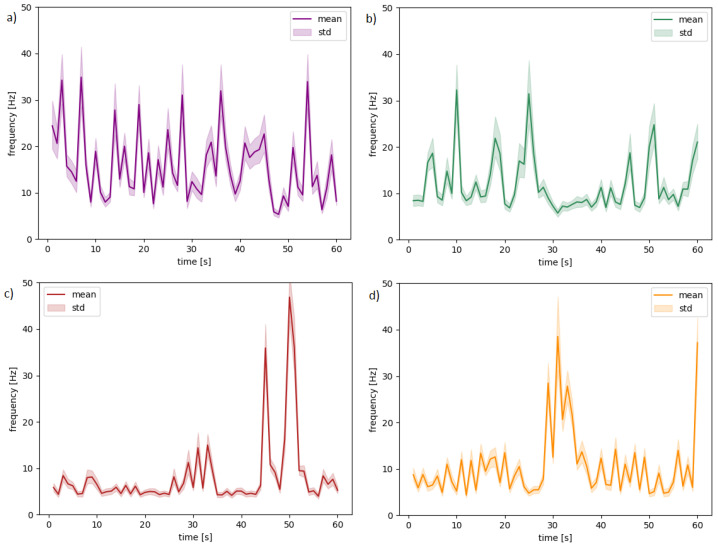
The waveforms for random subjects and different channels: (**a**) 11-T7 (**b**) 11-CP1, (**c**) 30-F7 (**d**) 30-CP5.

We propose choosing the commonly used mean statistics for every second of the recordings. The sample signal waveforms for four randomly selected subjects, videos, and channels are presented in Figure 4 to show the variability of the frequency values while watching a video. The solid lines in charts visualize the means, and light areas show standard deviation (variability between frequencies). Table 2 presents descriptions for each chart.

As a result, after preprocessing our data, we obtained 128 band-channel combinations using a frequency branch—32 electrodes × 4 bands.

### 3.4. Automated Feature Selection for Band-Electrode Combinations

Feature selection algorithms enhance computation time and classification accuracy and identify the most appropriate channels for a specific application or task. As it was introduced in Section 2, the process of channel selection might be crucial. Hence, the researchers developed new techniques for selecting the optimal number and location of electrodes.

Regarding one of the main EEG classification issues, i.e., redundancy, we suggested using the Reversed Correlation Algorithm (RCA). The algorithm uses correlation coefficients but in reverse order. It means that the RCA suggests features that are the least connected with all their predecessors. For that reason, the RCA might be beneficial in the reducing EEG signals’ redundancy.

The algorithm was proposed by Wosiak and Zakrzewska in [24]. The entire process is explained in Algorithm 1.
**Algorithm 1.** Reversed Correlation Algorithm1: **function** RCA(*N*)       ▹ N is a desired number of channels from R subset2:   $//Ch={ch}_{1},{ch}_{2},\ldots ,{ch}_{128}$          ▹ set of all channels (features)3:   $R_{1}\leftarrow$ take the first channel with the min correlation4:   **while**
i<N
**do**5:     **while**
*j* < length of Ch
**do**6:       **while**
*k* < length of *R*
**do**7:         value← compute correlation between channels8:         sum←sum + value9:         k←k+110:       **end while**11:       $R_{i}\leftarrow sum/len\left(R\right)$12:       sum←013:       j←j+114:     **end while**15:     $R_{i}\leftarrow$ choose channel with the lowest sum of value16:     k←k+117:   **end while**18:   **return**
$R_{i}$                   ▹ selected subset of channels19: **end function** 

The main goal is to find the channels that are the least correlated with each other. Every channel is divided into four main frequencies—theta, alpha, beta, gamma. Therefore, as an input, we have 128 features (32 channels × 4 frequencies), which represent the Ch subset. The first step of RCA is to enter the number of desired channels. The first band-channel with the lowest average correlation to the rest of the channels is calculated. The channel number is saved to the R subset (as $R_{1}$). Then, the loop starts, and we look for the next band-channels until obtaining N number from the input. The second band-channel is chosen as a value with the lowest correlation to the first attribute from R subset and is saved as an $R_{2}$. Another loop designates the next channel, wherein:

- correlation with each band-channel from R subset ($R_{1}$, $R_{2}$, etc.) is calculated for each band-channel from Ch subset, and
- the values are summed.

The loop ends when the number of channels for the *R* subset is obtained. In the next step, the sum is divided by the length of *R* list, and the channel number with the lowest sum is saved. The operation is repeated for every band-channel from the Ch subset. The lowest value is taken along with the number of that channel saved as the next channel in the *R* subset. The sum value is equal to 0, and we proceed to the next sub-channel. The algorithm ends when the length of subset *R* is equal to *N* input number.

We set the number of channels selected to three, according to other studies and data limitations. In order to compare the results of our channel selection by the RCA algorithm, we applied three other methods of electrode selection:

- based on mean correlations of the valence and arousal in all participants, as described by Koelstra et al. in [10],
- based on local subset feature selection and modified Dempster–Shafer theory as proposed by Soroush, Maghooli et al. in [23],
- based on three central electrodes, as described in [42].

### 3.5. Evaluation Criteria

Selecting an optimum subset of EEG channels requires an evaluation criteria. Concerning emotion classification, classifier-based measures may be used. A classifier is responsible for finding the separability measure for different classes described by labels, and a channel is selected when the classifier performs well. The Support Vector Machine (SVM) is among the most fundamental and popular classification methods. It was proposed by Cortes and Vapnik [56] and is based upon the principle of structural risk minimization. The SVM uses core functions to create a plane that can carry datasets, which are difficult to separate linearly into a high-dimensional space and maximizes the space between classes. Scientific studies reveal that the SVM contributes to better classification accuracy than other algorithms with feature vectors obtained from EEG signals [57,58]. A Gaussian radial basis function (RBF) kernel was used to enhance the data separability [55,59,60].

Classification values obtained for the selected subset of sensors were compared in terms of statistical significance. By significant differences, we assume *p* value ≤0.05.

## 4. Results and Discussion

The purpose of the experiments was to indicate a subset of EEG channels, which reduces the complexity of the analysis and signal redundancy and positively influences emotion classification.

The experiments were conducted according to the methods introduced in Section 3 on the dataset described in Section 3.2. Only valence and arousal were analyzed. Three steps of experiments were performed:

1. The experimental procedure of emotions’ classification that incorporated all 32 EEG electrodes to determine the possibilities of automatic feature selection methods in EEG signal extraction in terms of emotion recognition—the baseline for subsequent procedures.
2. The experimental procedure of emotion classification that uses the proposed RCA algorithm for EEG channel selection.
3. The experimental procedure of emotion classification that uses channels recommended in the literature: Koelstra et al. [10], Soroush et al. [23], and Frantzidis et al. [42].

The procedures were conducted with 10-fold cross-validation. The first step is to divide all data into ten subsets, which consist of independent trials. Each trial consisted of mean statistics for every second of one-minute recording, according to other relevant studies [10,23,42,61,62]. One of these subsets is selected as test data, and the others are summed and used for training. The process is repeated ten times and returns the average accuracy and standard deviation. Both training and testing sets were formed by an inter-user approach, i.e., summing up all subjects’ EEG data and shuffling it. The RCA algorithm is applied on the training set, and the selected features are used as a reference over the testing set. The process for training and testing data was introduced in Figure 2. The experimental environment was based on the Python programming language and its libraries.

The results of the experiments were gathered in Table 3 and Table 4. Table 3 presents the results of channel selection, where each row represents a particular feature selection approach. The fourth column contains the names of selected channels along with bands of frequencies, and the last column illustrates the electrodes’ locations.

It can be seen in Table 3 that several methods, including the proposed RCA, selected Cz electrode, which is consistent with the perspective of psychology. The study described in [63] emphasized the strong activity of the Cz electrode associated with emotional states. We can also notice that apart from the central lobe, most of the methods (i.e., CHS_RCA, CHS1, CHS2, CHS3, CHS4, CHS5) favor the right part of the brain. It can by justified by two psychological factors, namely:

- the studies show that higher left-brain activity is related to a positive emotional state, while higher right-brain activity reflects a negative emotional state [41],
- negative emotions are generally believed to elicit more reactivity [39,40,64].

The analysis of frequency bands selected by the methods presented in Table 3 revealed that CHS3, CHS4 and CHS6 focused on the Beta band, whereas CHS7 included only the Theta band. Our proposed method CHS_RCA distinguished different bands. These findings can be useful in emotion differentiation, as according to the studies [65,66,67]:

- the lateral temporal areas are mostly stimulated by positive emotions in beta and gamma bands,
- the neural patterns of neutral emotions have higher alpha responses at parietal and occipital sites,
- the neural patterns have significant higher delta responses at parietal and occipital sites and higher gamma responses at prefrontal sites for negative emotions.

Table 4 describes classification results for RCA channel selection compared to classification based on all electrodes and three other solutions, as introduced in Section 3.4. It is worth mentioning that some of the channel selection methods were related to a particular emotion, and therefore, some of the corresponding cells in Table 4 may be left blank (—). The third and the sixth column of Table 4 highlight the difference between classification accuracy attained by the subset of channels pointed by the proposed RCA algorithm and the corresponding channel selection method.

One can notice that the RCA outperformed other channel selection methods in terms of valence and arousal classification. Moreover, most of the outperformed differences were statistically significant (*p* value ≤0.05). The RCA algorithm returned worse results only in three out of twelve comparisons, wherein none of those differences was statistically significant. What is more, the CH2 method—three channels proposed by Koelstra et al. in [10] were adjusted to only one type of emotion classified, whereas our method may deal with different types of emotions.

It is also worth emphasizing that our RCA algorithm is an unsupervised machine learning technique, which is an advantage in terms of uncertain or unlabeled data. The variability in emotion expression and the subjectivity of emotion perception may lead to datasets with missing or uncertain labels. Many attempts have been undertaken to solve this problem, with the application of unsupervised methods of machine learning being among the most popular [68,69].

It can be also noted that the attained classification results of emotions may be perceived as inferior in comparison to works related to EEG signal analysis in other domains [46]. However, it must be stressed that efficient extraction of features for emotion classification is more complex as emotions are less predictable and less explainable by EEG signals [70].

To conclude, the presented results of the study are very encouraging. However, in the field of emotion classification and EEG analysis, there is still abundant room for further analysis. Firstly, we performed our experiments on only one dataset. Although the DEAP dataset is a primary source for studies related to emotion recognition [10,23,55,60,71], multiple datasets [72,73] will be used in further research to prove the significance of the proposed method. Secondly, deeper insight into signal decomposition methods should be considered, as they play an important role in BCI applications [74,75,76,77]. Our approach involved traditional signal denoising [10]. However, other denoising methods are worth considering, as the studies on multiscale component analysis (MSPCA) proved to have very good results in terms of classification accuracy for motor imagery applications [78]. The existing research studies have also confirmed that the combination of signal decomposition with dimensionality reduction techniques along with neural networks provide good accuracy for subject-dependent and -independent motor imagery based systems [44] and for emotion recognition solutions [79]. Therefore, future research may also explore neural networks and deep learning approaches. Moreover, data augmentation by sliding windows will be considered, as it is increasingly used with deep learning on EEG [80].

## 5. Conclusions

The proposed research addresses the problem of finding correlations between human emotions and the activated brain regions based on EEG signals. Optimal EEG channel selection and location is a challenging task due to the interconnections between electrodes. The proposed hybrid method incorporating the RCA algorithm finds combinations of electrodes and their frequency bands, which are least correlated with one another, and therefore, enables redundancy reduction and classification advancement. The obtained classification accuracy results for two emotions—valence and arousal—were, on average, 4.20–8.44% greater than channel selections suggested in different works.

Our method based on the Reversed Correlation Algorithm is simple to use. At the same time, it significantly reduces the number of data needed for the analysis. As a method of unsupervised machine learning techniques, it can also be used for unlabeled data or datasets where the obtained labels are uncertain.

Nonetheless, there is still a need for additional study. Therefore, further research is planned to investigate multiple datasets. Moreover, deeper insight into denoising methods and decomposition in terms of frequency-domains and time-frequency domains is planned. Future research may also explore neural networks and deep learning approaches to find unknown attribute correlations.

## Figures and Tables

**Table 1:** Five Bands for Brain Waves.

EEG Band	Frequency Range [Hz]	Brain State
delta (δ)	0.5–3.5	Deep sleep
theta (θ)	4–7	Mental relaxation
alpha (α)	8–12	Clear-headed, passive attention
beta (β)	13–30	Brain awakening and alertness, active, external attention
gamma (γ)	31–47	High frequency, Concentration

**Table 2:** Channel allocation distribution to Figure 4.

Figure ID	Channel Name	User ID	Video ID
(a)	T7	11	17
(b)	CP1	11	9
(c)	F7	30	5
(d)	CP5	30	28

**Table 3:** Channel selection.

Method ID	Authors	Emotions	Channels	Locations
CHS_RCA	Wosiak et al.	Valence/Arousal	P8 Alpha AF4 Gamma Cz Theta	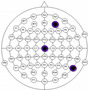
CHS1	Koelstra et al. [10]	Valence	PO4 Theta FC6 Beta Cz Beta	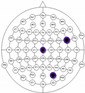
CHS2	Koelstra et al. [10]	Arousal	CP6 Theta Cz Alpha FC2 Beta	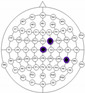
CHS3	Koelstra et al. [10]	Valence	FC2 Beta FC6 Beta Cz Beta	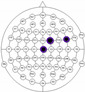
CHS4	Soroush et al. [23]	Arousal	PO4 Beta FC1 Beta FC6 Beta	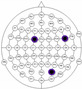
CHS5	Soroush et al. [23]	Valence	FC6 Beta P4 Theta PO4 Theta	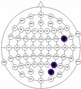
CHS6	Frantzidis et al. [42]	Valence/Arousal	Cz Beta Fz Beta Pz Beta	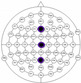
CHS7	Frantzidis et al. [42]	Valence/Arousal	Cz Theta Fz Theta Pz Theta	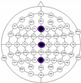

**Table 4:** Results of classification accuracy [in %].

	Valence			Arousal		
Method ID	ACC	RCA Gain	*p*-Value	ACC	RCA Gain	*p*-Value
CHS_RCA	74.00	—	—	74.00	—	—
None	49.81	+24.19	<0.001	62.41	+11.59	<0.001
CHS1 [10]	70.55	+3.45	0.079	—	—	—
CHS2 [10]	—	—	—	74.18	−0.18	0.487
CHS3 [10]	75.33	−1.33	0.291	54.80	+19.20	0.003
CHS4 [23]	54.72	+19.28	<0.001	—	—	—
CHS5 [23]	—	—	—	61.67	+3.58	0.019
CHS6 [42]	66.25	+7.75	<0.001	80.41	−6.41	0.131
CHS7 [42]	60.94	+13.06	<0.001	69.17	+4.83	0.196
